# The Book World of Medicine and Science

**Published:** 1902-12-13

**Authors:** 


### The Book World of Medicine and Science.

The Theory and Practice of Infant Feeding. By Henry Dwight Chapin, A.M., M.D. (London: Baillière, Tindall and Cox. 1903. Price 10s. 6d.).

We have read Dr. Chapin's book on the theory and practice of infant feeding with considerable interest, and we believe with no little profit to ourselves. It possesses the great merit of approaching this favourite and, we had almost believed, threadbare subject from standpoints which, if not exactly new, have heretofore been practically neglected, namely, from the sides of comparative physiology and biology. Many of us who follow the contemporary progress in this department of dietetics are as weary of attempts to measure in terms of calories the food requirements of the developing child as we are impatient with those vain theorisers who believe there is only one royal road to success in the dietetics of infancy, and that this road is their own particular property. It has been wisely said that an infant is a law unto itself; he who attempts to systematise or codify these individual laws in the form of a universal jurisprudence labours in vain. Dr. Chapin insists that physiological chemistry is not in a position to explain the differences between the milks of different species of the animal series. The milk of each animal is adapted to the digestive system, the rate of growth, and the nutritive requirements of its own particular offspring, and on biological grounds it is improbable that we shall ever find any exact equivalent, although on chemical and physical grounds many of the substitutes which have been provided present no material differences. The only scientific method of approaching the subject of infant feeding is, according to Dr. Chapin, to study the life history not only of that "commonwealth of cells," namely, the child himself, but also of the individual cells of which the commonwealth is constituted. Further, it is necessary to possess a knowledge of the food material which is needed for the growth and maintenance of the cells, and to investigate the means whereby the requisite food material may reach the cells and be assimilated by them.

Starting on the sound basis that cow's milk is not human milk and never can be, the author proceeds to instruct his readers on the best method of modifying the former so as to resemble the latter physiologically, chemically and physically. Like all other sound pediatrists, Dr. Chapin is a believer in percentage feeding, for the simple reason that no other method contains even the elements of accuracy; he has, however, little to say in favour of leaving the preparation of percentage mixtures to the officials of a milk laboratory; in spite of its incidental and necessary inaccuracies he would trust rather to a home method of modification, and on this point we find ourselves in entire agreement with him. As long as it is possible to repeat on the morrow the performances of the day which has passed and to alter in any required direction the percentages or the relative proportions of the contained elements of a milk mixture, it is of comparatively little importance to know whether the percentage of proteid, for instance, is 1 per cent. or 1·5 per cent. The test of the suitability of a food is not the absolute chemical analysis, but the biological or physiological reaction of the recipient. Variations in the relative proportions of the important elements of milk can be controlled in a practical and efficient manner by the method which the author recommends, namely, by the utilisation and subsequent dilution of different amounts of the upper layers of milk which has been allowed to stand for some hours in a graduated bottle. It has been proved practically that in four hours all the cream contained in the milk will have risen to the upper third. By withdrawing by means of the author's dipper the upper portions down to various levels, and by diluting the top milk thus obtained with water, a mixture containing almost any requisite percentage of fat and proteid can be readily obtained. The only objection to this method is the necessary delay which is involved in allowing the milk to stand for four hours. Personally we regard this method as less satisfactory than the method of mixing milk with various proportions of cream of a known percentage, although we are aware that many authorities prefer the method which Dr. Chapin himself employs. We are glad to notice that the author lays great stress on the evil results which are likely to accrue from feeding infants on an excess of sugar and a deficiency of proteid. The experiments which he quotes to prove the bad quality of bones, muscles, and other tissues which develop under such injurious methods of feeding should be sufficient to convince all those, and we are afraid there are many of them who do not know the disastrous consequences of such mistakes in feeding. To sum up, the main conclusions which are to be drawn from this most excellent and scientific handbook of infant feeding:—(1) Although cow's milk differs materially from human milk, if properly modified it is the best substitute for maternal feeding. (2) The natural and biological properties of milk must be interfered with by chemical and physical means as little as possible. (3) For the growth of sound, resistant, healthy tissues an adequate percentage of proteid elements in food is absolutely essential.

In conclusion we would strongly recommend all those who are interested in the theory and practice of infant feeding to obtain Dr. Chapin's illuminating book and read it carefully.

The Care of the Skin and Hair: Containing Suggestions as to Diet, Clothing, Bathing, and Cosmetics. By James Startin, M.R.C.S. (Bristol: John Wright and Co. 1902. Price 2s. 6d. net.)

A pretty little book, bound in white and gold, easy to read, and containing a good deal of information on the subject which it deals with. This is a work which is apparently addressed to the laity. We are not fond of such books. On questions of diet and clothing, however, on baths and bathing, on the use and abuse of cosmetics, and on the care of the hair, the advice given seems to have been carefully considered; but there is nothing in it which is likely to be of interest to medical practitioners.

### BOOKS RECEIVED.

C. Griffin and Co.

"Official Year Book of the Scientific and Learned Societies of Great Britain and Ireland, 1902."

McCorquodale and Co., Limited.

"Metropolitan Asylums Board Annual Report, 1901." 2 vols.

J. B. Lippincott Co.

"International Clinics." Vol. iii. Twelfth series.

The Army and Navy Gazette.

"Mentioned in Despatches." Complete list. Second Edition.

Baillière, Tindall and Cox.

